# Safe Corridor to Access Clivus for Endoscopic Trans-Sphenoidal Surgery: A Radiological and Anatomical Study

**DOI:** 10.1371/journal.pone.0137962

**Published:** 2015-09-14

**Authors:** Ye Cheng, Siwen Zhang, Yong Chen, Gang Zhao

**Affiliations:** 1 Department of Neurosurgery, First Hospital of Jilin University, Changchun City, Jilin Province, P. R. China; 2 Department of Endocrine, First Hospital of Jilin University, Changchun City, Jilin Province, P. R. China; Emory University School of Medicine, UNITED STATES

## Abstract

**Purpose:**

Penetration of the clivus is required for surgical access of the brain stem. The endoscopic transclivus approach is a difficult procedure with high risk of injury to important neurovascular structures. We undertook a novel anatomical and radiological investigation to understand the structure of the clivus and neurovascular structures relevant to the extended trans-nasal trans-sphenoid procedure and determine a safe corridor for the penetration of the clivus.

**Method:**

We examined the clivus region in the computed tomographic angiography (CTA) images of 220 adults, magnetic resonance (MR) images of 50 adults, and dry skull specimens of 10 adults. Multiplanar reconstruction (MPR) of the CT images was performed, and the anatomical features of the clivus were studied in the coronal, sagittal, and axial planes. The data from the images were used to determine the anatomical parameters of the clivus and neurovascular structures, such as the internal carotid artery and inferior petrosal sinus.

**Results:**

The examination of the CTA and MR images of the enrolled subjects revealed that the thickness of the clivus helped determine the depth of the penetration, while the distance from the sagittal midline to the important neurovascular structures determined the width of the penetration. Further, data from the CTA and MR images were consistent with those retrieved from the examination of the cadaveric specimens.

**Conclusion:**

Our findings provided certain pointers that may be useful in guiding the surgery such that inadvertent injury to vital structures is avoided and also provided supportive information for the choice of the appropriate endoscopic equipment.

## Introduction

The clivus is a region of the skull base extending from the posterior clinoid process (PCP) to the foramina magnum. It is formed by the corpora ossis sphenoidalis and the basilar part of the occipital bone and is closely associated with tumors originating from the ventral aspect of the brain stem [1]. Since the clivus is situated deep within the recesses of the skull and it is closely associated with several important neurovascular structures, such as the brainstem, pituitary, and internal carotid artery, it is difficult to access using conventional surgical methods; the bony barrier to the arteriae vertebralis is another obstacle to the access of the clivus [2]. The rapid technological advances in endoscopic surgery have led to the introduction of the extended trans-nasal trans-sphenoidal approach for the effective surgical removal of tumors in the sellar region and the ventral aspect of the brain stem [3–4]. However, this surgical approach is made complex by the requirement of comprehensive anatomical understanding of the area to accurately identify the site of surgical resection and avoid the complications of injury to the adjacent neurovascular structures. With this background, we sought to undertake a novel radiological and anatomical study of the clivus and important adjacent neurovascular structures that are relevant to the extended trans-nasal trans-sphenoidal approach.

## Materials and Methods

### Study design

This study was designed as a retrospective investigation of the anatomical features of the clivus and related structures by using computed tomographic angiography (CTA) images obtained from 220 adults between January 2013 and December 2013. The radiological data were obtained from the electronic record system maintained at the workstation of the Radiology Department of our institution. The data from the individuals were analyzed anonymously, and the study protocol was approved by the Ethics Committee of First Hospital of Jilin University. Patient records were anonymized and de-identified prior to the analysis.

Among the 220 adults, 118 were male and 102 were female, and the ages of the subjects ranged from 18 to 80 years (mean age, 49.9 years). All the computed tomography angiography (CTA) images were obtained by using the same device, 64-multidetector row spiral CT scanner (Siemens Healthcare, Germany; slice thickness, 0.625 mm) at the outpatient clinic of the First Hospital of Jilin University. Images that showed evidence of sphenoid sinus disease, internal carotid artery (ICA) malformations, clivus fracture, and space-occupying lesions (29 of the total 249) were excluded from the study.

Multiplanar reconstruction (MPR) of the CT images was performed, and measurements were made in the coronal, sagittal, and axial planes. In addition, magnetic resonance (MR) image of 50 adults were examined to determine the position of the origin of the trigeminal nerve, since this is an important structure likely to be injured in surgeries of the brain stem. Moreover, 10 dry skulls of adult cadavers obtained from the department of human anatomy of Norman Bethune Health Science Center at our University were examined to compare the measurements obtained using the CT images in this study and verify their practicability.

**Fig 1:**
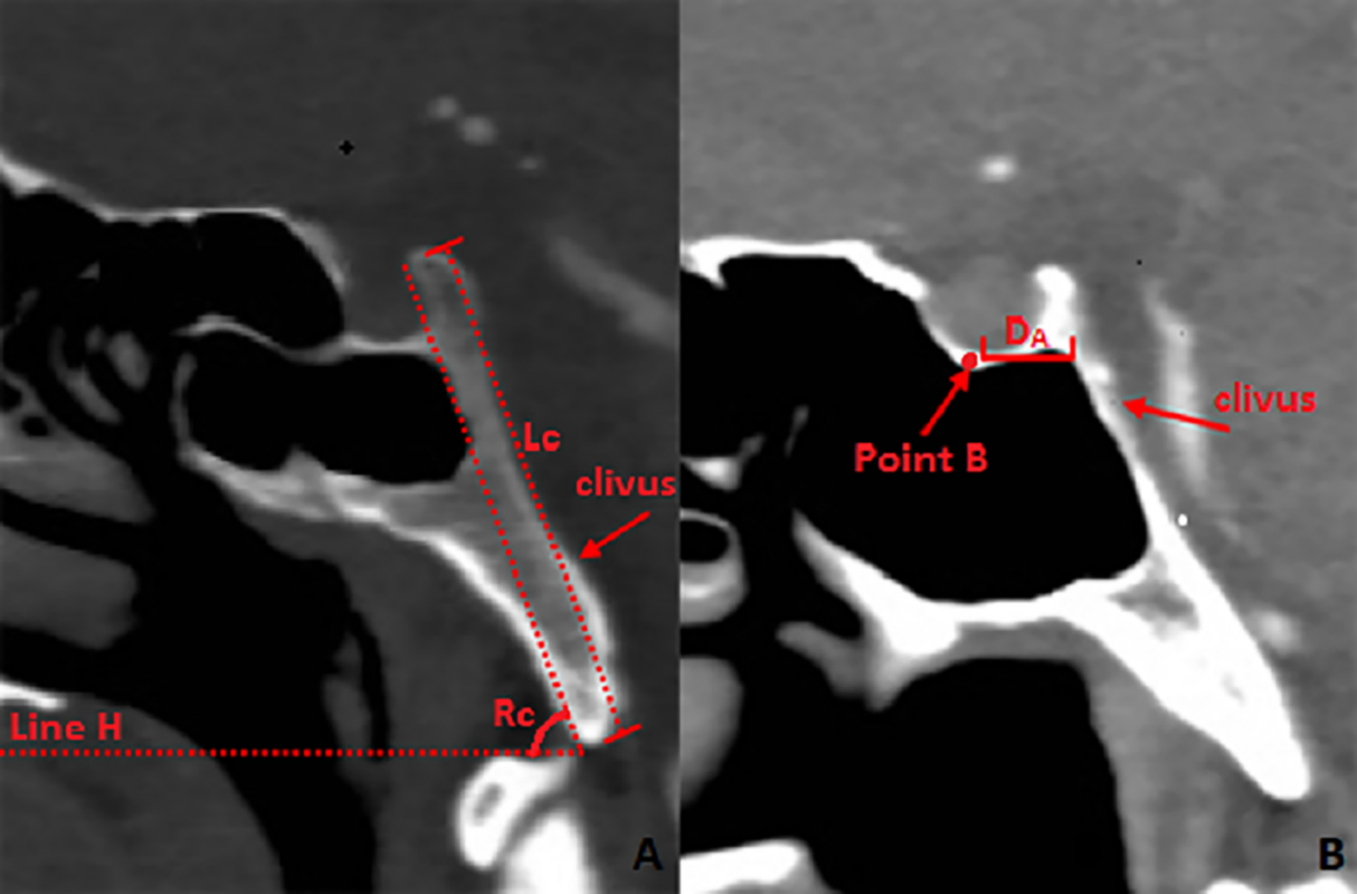
Measurement of the clivus in the sagittal plane of the CT image. **1A:** Measurement of the length and angle clivus in sagittal plane in CT image (bone window); **Rc: **The angle between the clivus and the horizontal plane; **Lc:** the length of the clivus; **Line H:** The horizontal line. **1B:** Distance from the lowest point of the sellar floor (SF) to the clivus and vertebral artery in CT image (soft-tissue window): **D**_**A**_: The horizontal distance from the lowest point of SF to the posterior boundary of the clivus; **Point B:** The middle lowest point of the SF.

**Fig 2:**
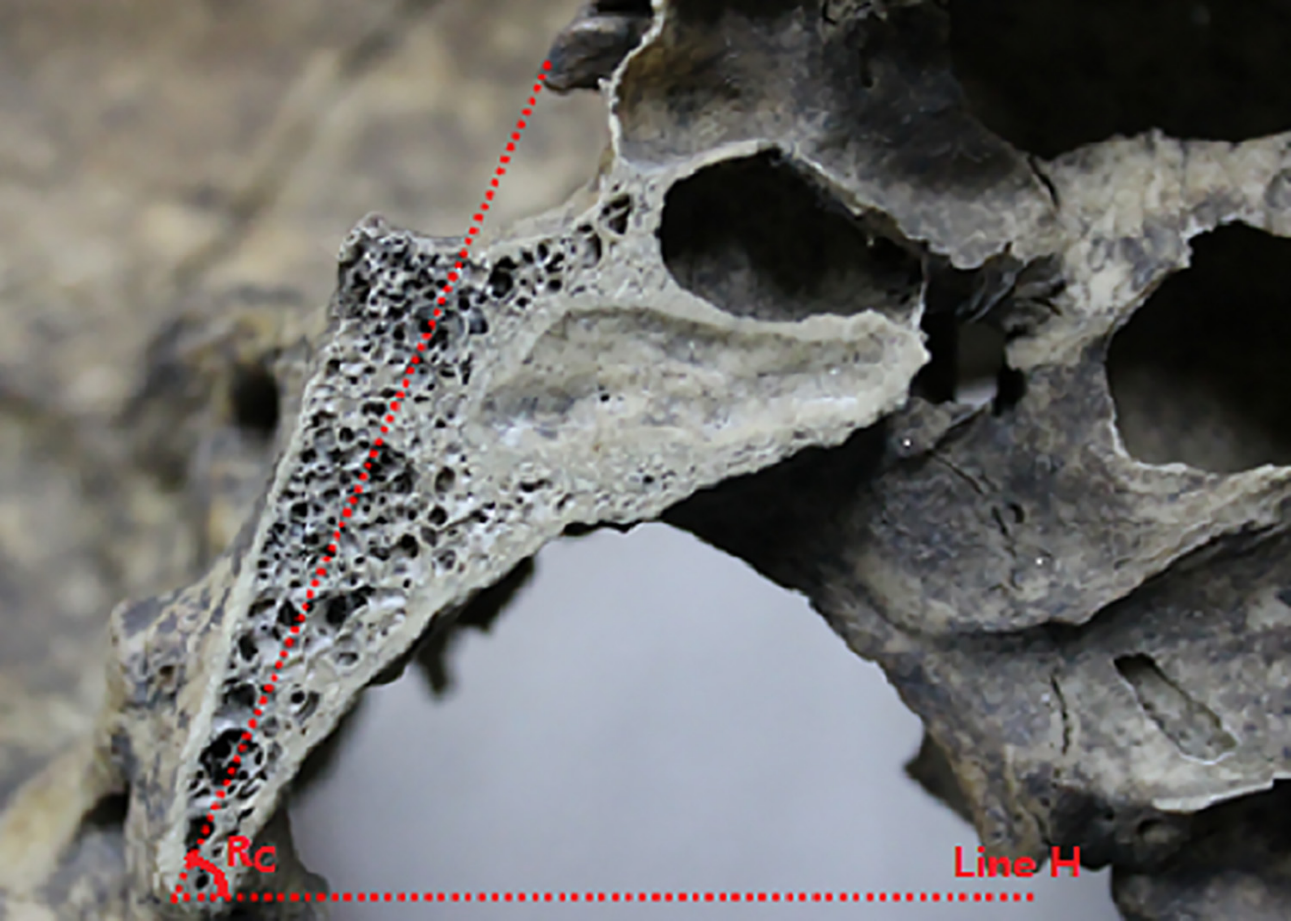
Measurement of the angle of clivus in sagittal plane in skull specimen. **Rc:** The angle between the clivus and the horizontal plane; **Line H:** The horizontal line.

**Fig 3:**
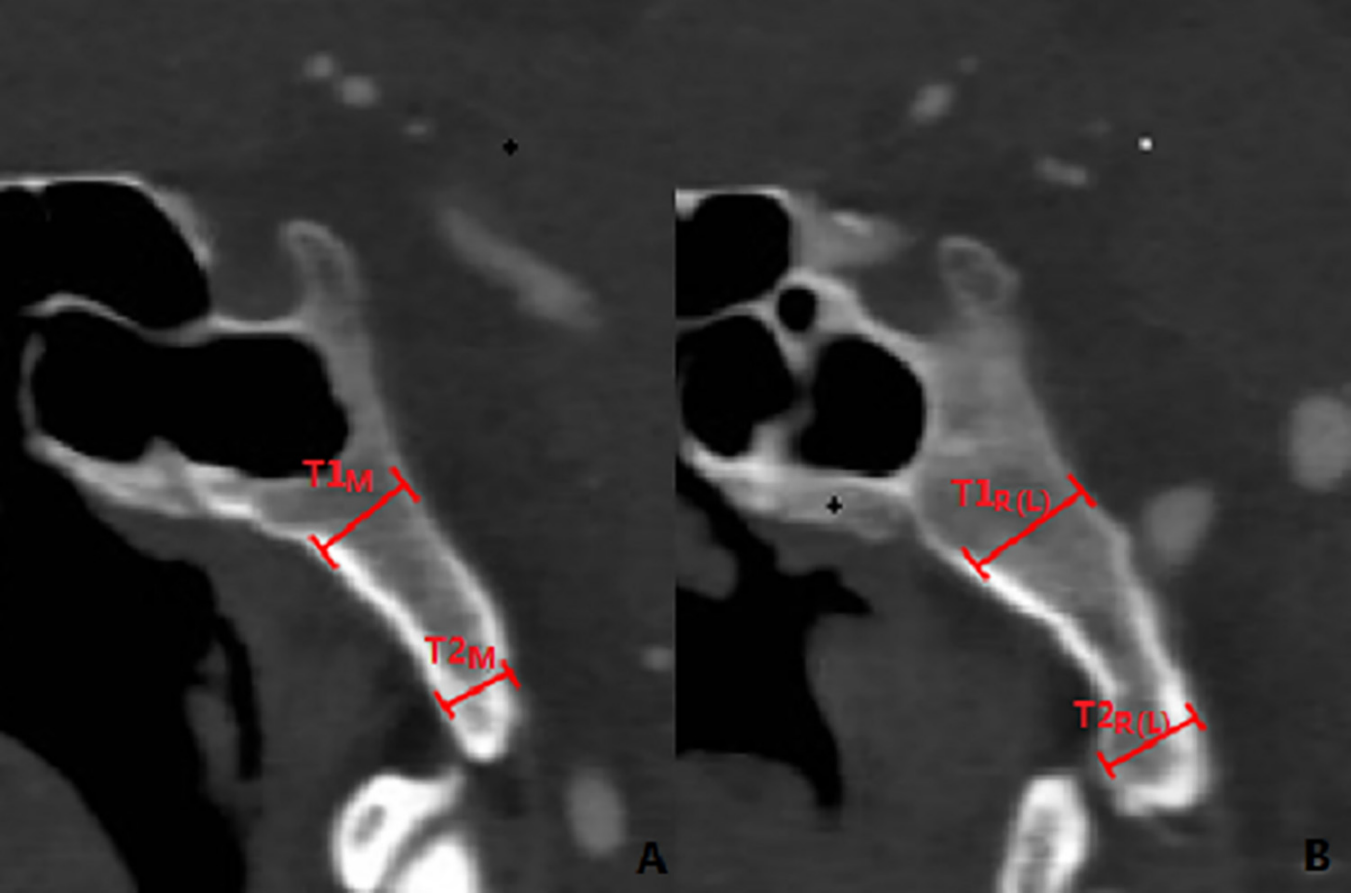
Thickness of clivus. **3A:** Measurement of the thickness of the clivus in the mid-sagittal plane: **T1**_**M**_: the thickness of the middle portion of the clivus in the middle; **T2**_**M**_: the thickness of the inferior portion of the clivus in the middle; **3B:** Measurement of the thickness of the clivus in sagittal plane through the posterior nares: **T1**_**R (L)**_: the thickness of middle portion of the clivus in the right (left); **T2**_**R (L):**_ the thickness of the inferior portion of the clivus in the right (left).

**Fig 4:**
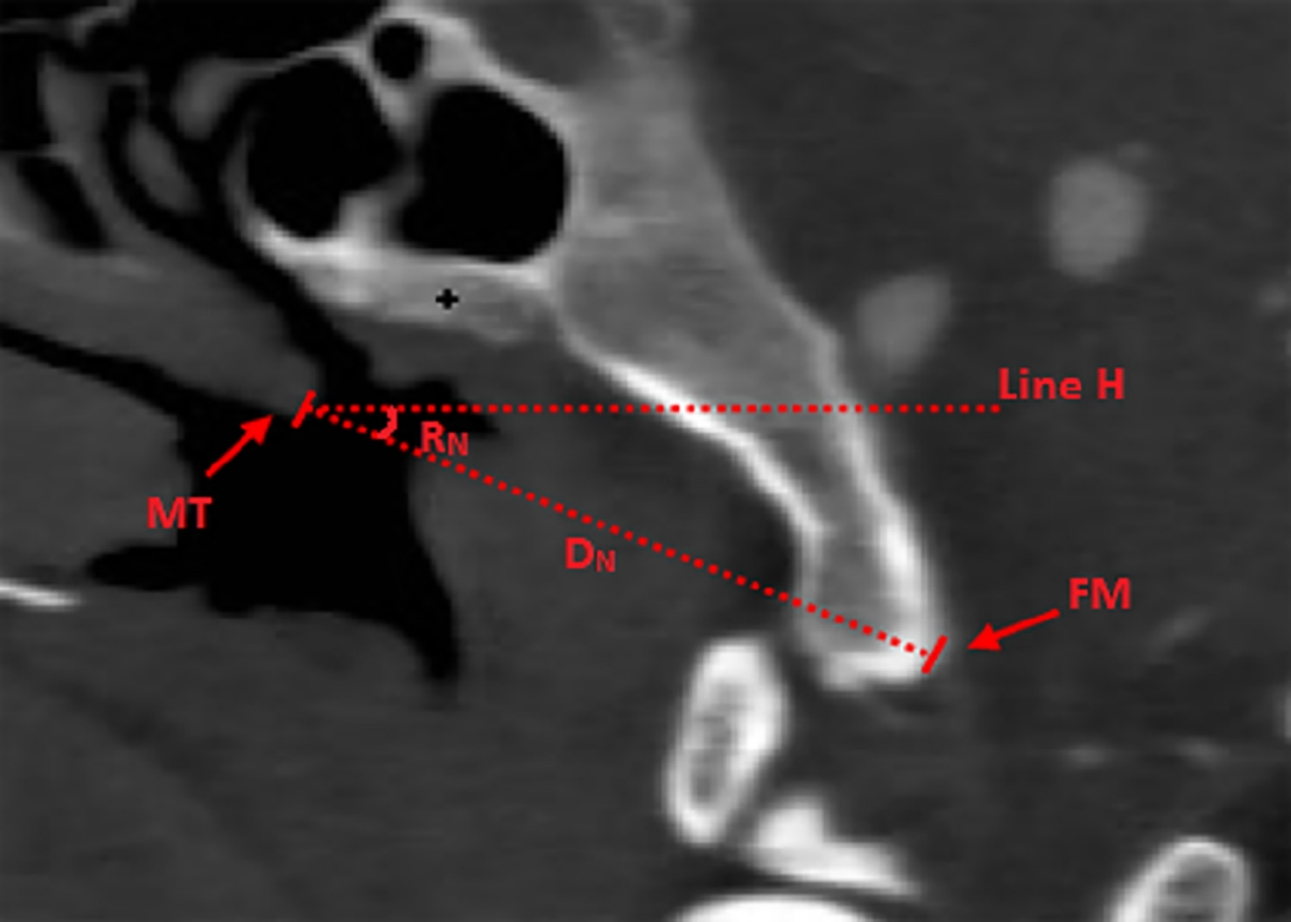
The distance between the middle turbinate (MT) and anterior margin of foramen magnum. **D**_**N**_: The distance between the posterior point of MT and anterior margin of foramen magnum; **R**_**N**_: The angle between the line from the posterior point of MT to anterior margin of foramen magnum and the horizontal plane; **Line H:** the horizontal line; **MT:** middle turbinate; **FM:** foramen magnum.

**Fig 5:**
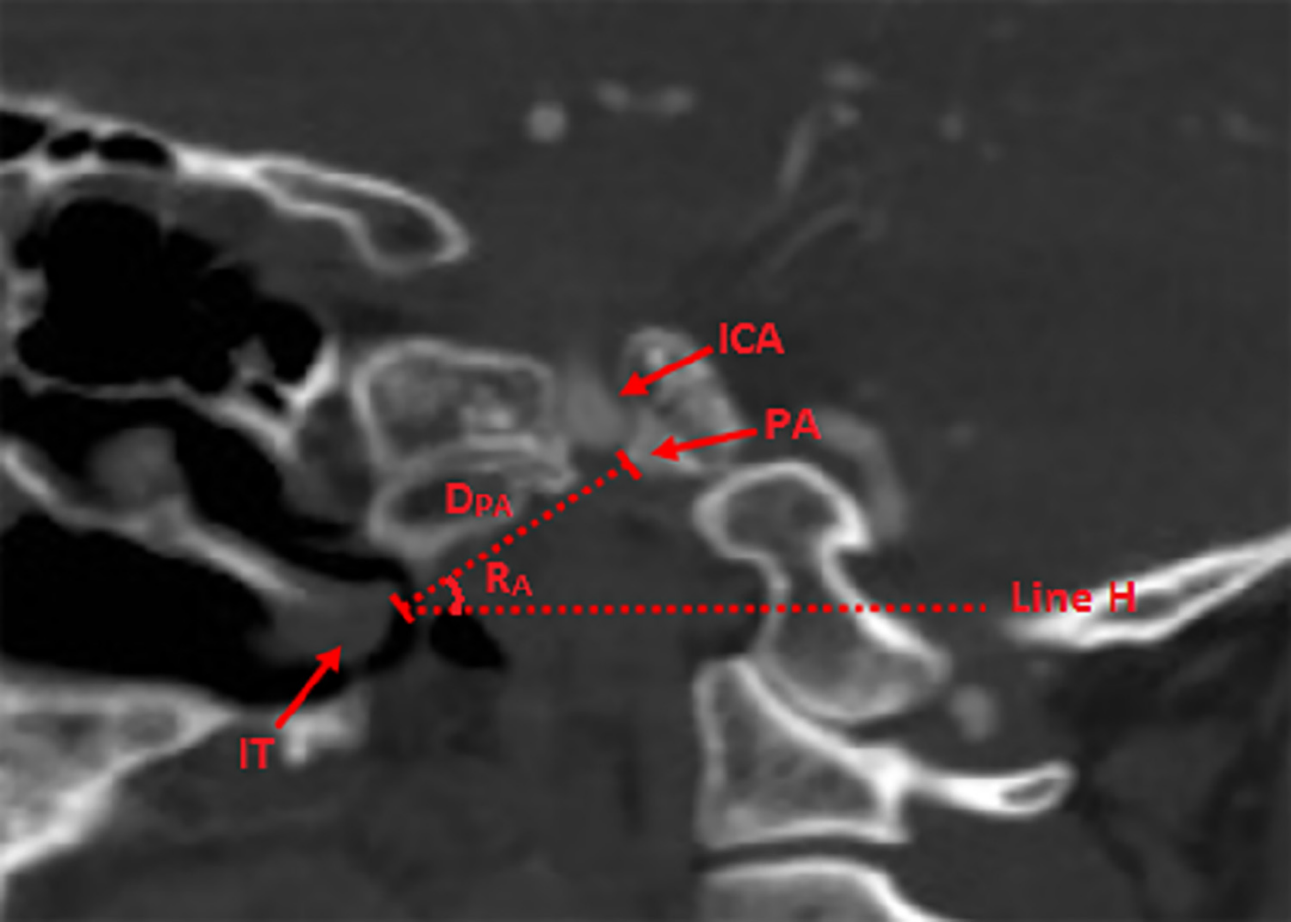
Location of petrous apex (PA) by the posterior point of inferior turbinate (IT) in the sagittal plane. **D**_**PA**_: Distance between the posterior point of IT and PA; **R**_**A**_: Angle between the line from the posterior point of IT to PA and the horizontal plane; **Line H:** the horizontal line; **IT:** inferior turbinate; **ICA:** internal carotid artery; **PA:** petrous apex.

**Fig 6:**
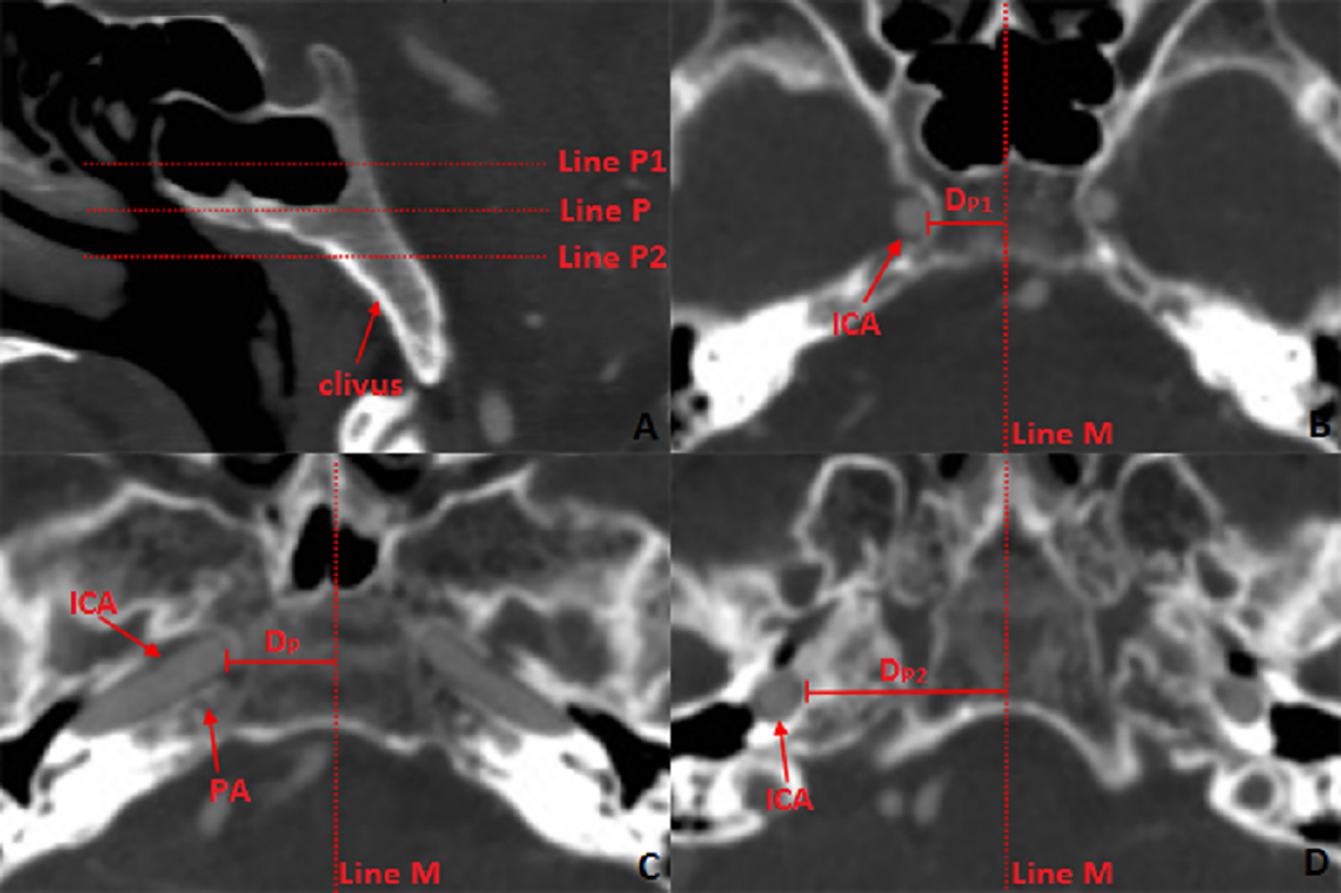
Distance between ICA and the mid-sagittal line in the position superior and inferior of the petrous apex (PA). **6A**: Position of the axial plane selected for the measurement: **Line P:** Position of the axial plane through PA; **Line P1**: Position of the axial plane 10 mm superior to Line P; **Line P2**: Position of the axial plane 10 mm inferior to Line P. **6B:** Axial plane 10 mm superior to PA. **Line M**: mid-sagittal line; **D**_**P1**_: distance between ICA and the sagittal middle line; **ICA:** internal carotid artery; **6C:** Axial plane through PA. **Line M:** mid-sagittal line; **D**_**P**_: distance between ICA and the sagittal middle line; **ICA**: internal carotid artery; **6D:** Axial plane 10 mm inferior to the petrous apex. **Line M**: mid-sagittal line; **D**_**P2**_: distance between ICA and the mid-sagittal line; **ICA**: internal carotid artery.

The mid-sagittal line passing through the sellar floor (SF) was identified by the central, lowest point on the pituitary fossa and optic recess, which is a consistent, symmetrical bony structure. The horizontal distance from the lowest point of the SF to the posterior boundary of the clivus was measured along the mid-sagittal plane (Fig 1). Further, the dry skulls were split along the mid-sagittal plane to measure the angle between the long axis of the clivus and the horizontal plane in order to account for the errors in measurements in the CT images due to the position of the subject in the CT scanner (Fig 2). Additionally, we measured the bone thickness of the clivus at the mid-sagittal plane and sagittal plane through the posterior nares (Fig 3) as well as the distance between the posterior point of the middle turbinate (MT) and anterior margin of the foramen magnum at the sagittal plane through the MT (Fig 4). We located the petrous apex (PA) by the posterior part of the inferior turbinate (IT) and analyzed the position of the paraclival carotid on both sides in the axial plane 10 mm inferior and superior to PA (Figs 5 and 6).

**Fig 7:**
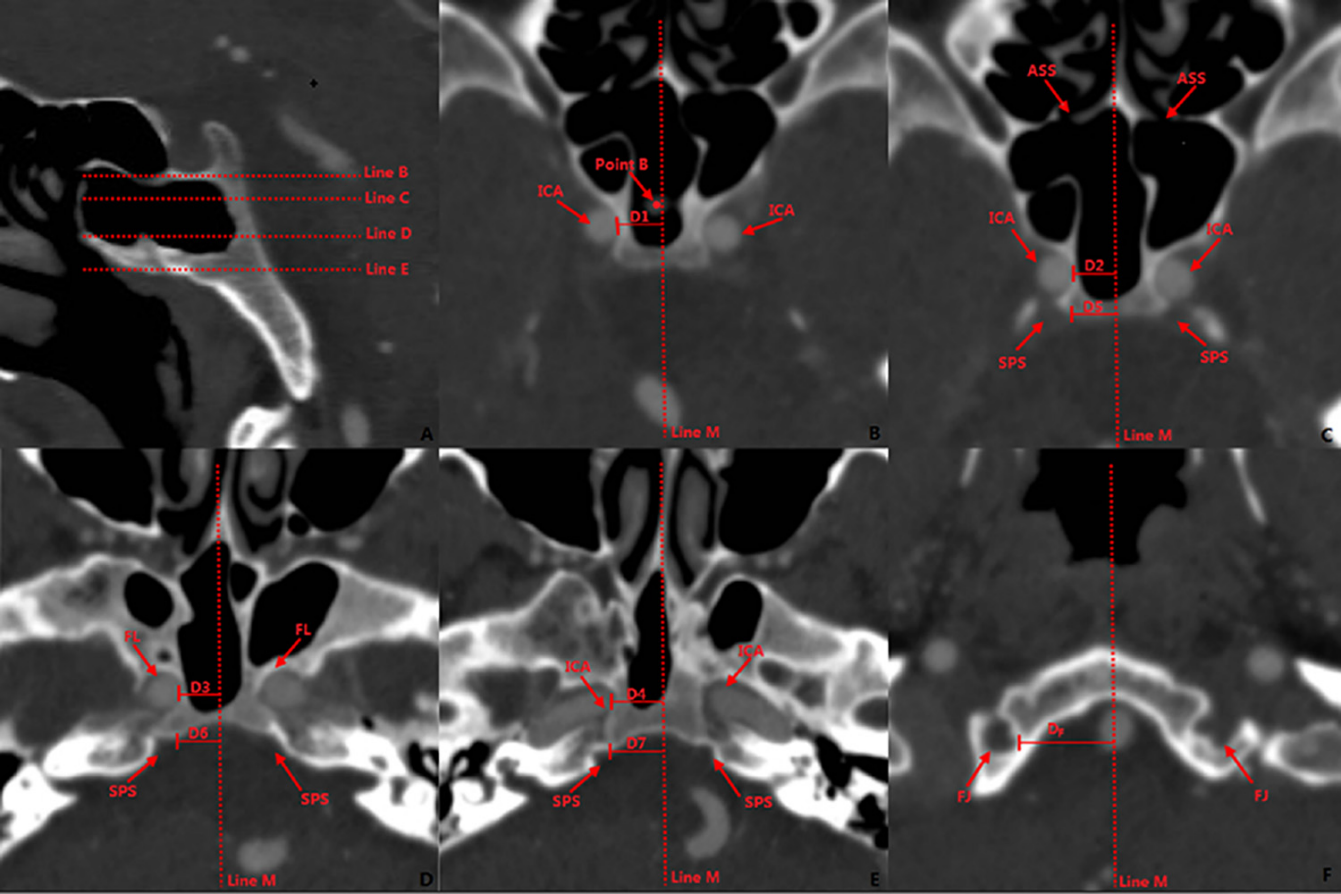
Measurement of safe region for penetration of the clivus. **7A:** Position of the axial plane selected for the measurement: **Line B:** Position of axial plane through middle lowest point of SF in Fig 7B; **Line C:** Position of the axial plane through apertura sinus sphenoidalis in Fig 6C; **Line D:** Position of the axial plane through the upper aspect of foramen lacerum in Fig 6D; **Line E:** Position of the axial plane passing through the superior margin of the nostril in Fig 6E. **7B:** Axial plane through the lowest central point of the SF: **Line M:** mid-sagittal line; **Point B:** lowest, central point of the SF; **ICA:** internal carotid artery; **D1:** distance between the medial wall of the internal carotid artery and mid-sagittal line. **7C:** axial plane through apertura sinus sphenoidalis: **Line M:** mid-sagittal line; **ASS:** apertura sinus sphenoidalis; **ICA:** internal carotid artery; **SPS**: sulcus of the inferior petrosal sinus; **D2:** Distance between the medial wall of the ICA and mid-sagittal line. **D5:** Distance between sulcus of the inferior petrosal sinus and the mid-sagittal line. **7D:** axial plane through upper of the foramen lacerum: **Line M:** the mid-sagittal line; **SPS:** sulcus of the inferior petrosal sinus; **FL:** foramen lacerum; **D3:** Distance between the medial margin of the foramen lacerum and mid-sagittal line; **D6:** Distance between the sulcus of the inferior petrosal sinus and the mid-sagittal line. **7E:** axial plane through the superior margin of the nostril: **Line M:** mid-sagittal line; **SPS:** sulcus of the inferior petrosal sinus; **D4:** Distance between the medial wall of the ICA and mid-sagittal line; **D7:** Distance between the sulcus of inferior petrosal sinus and the mid-sagittal line. **7F:** Distance between foramen jugulare to mid-sagittal line at SF. **D**_**F**_: Distance between the foramen jugulare to the mid-sagittal line at SF; **Line M:** the sagittal midline; **FJ:** foramen jugulare.

**Fig 8:**
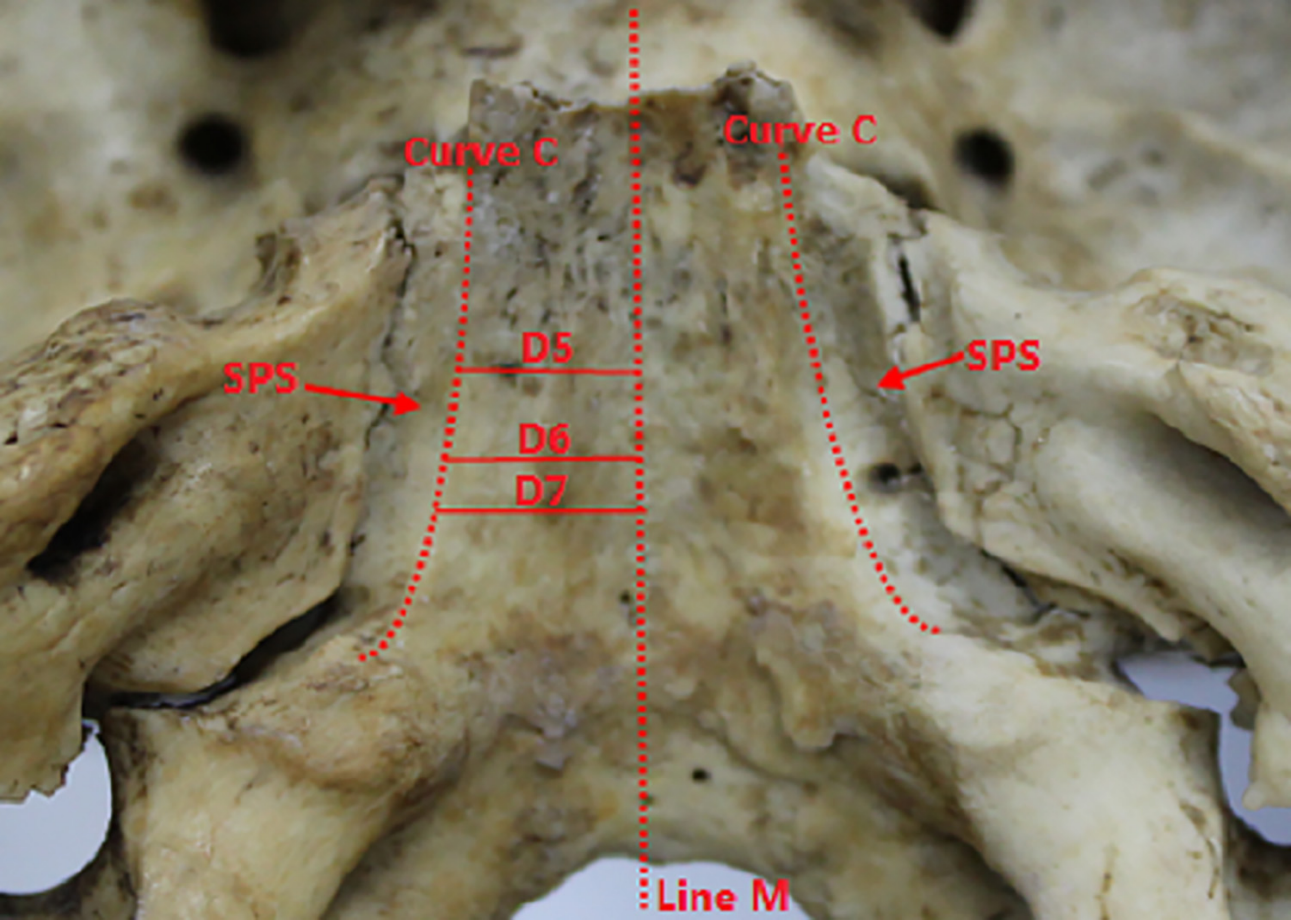
Sulcus of the inferior petrosal sinus measured in the specimen. **Line M:** Mid-sagittal line; **Curve S:** the medial boundary of the sulcus of the inferior petrosal sinus (safe corridor for fenestration of the posterior aspect of the clivus); **SPS:** sulcus of the inferior petrosal sinus; **D5:** Distance between the sulcus of the inferior petrosal sinus and the mid-sagittal line; **D6:** Distance between the sulcus of the inferior petrosal sinus and mid-sagittal line; **D7:** Distance between the sulcus of the inferior petrosal sinus and mid-sagittal line.

**Fig 9:**
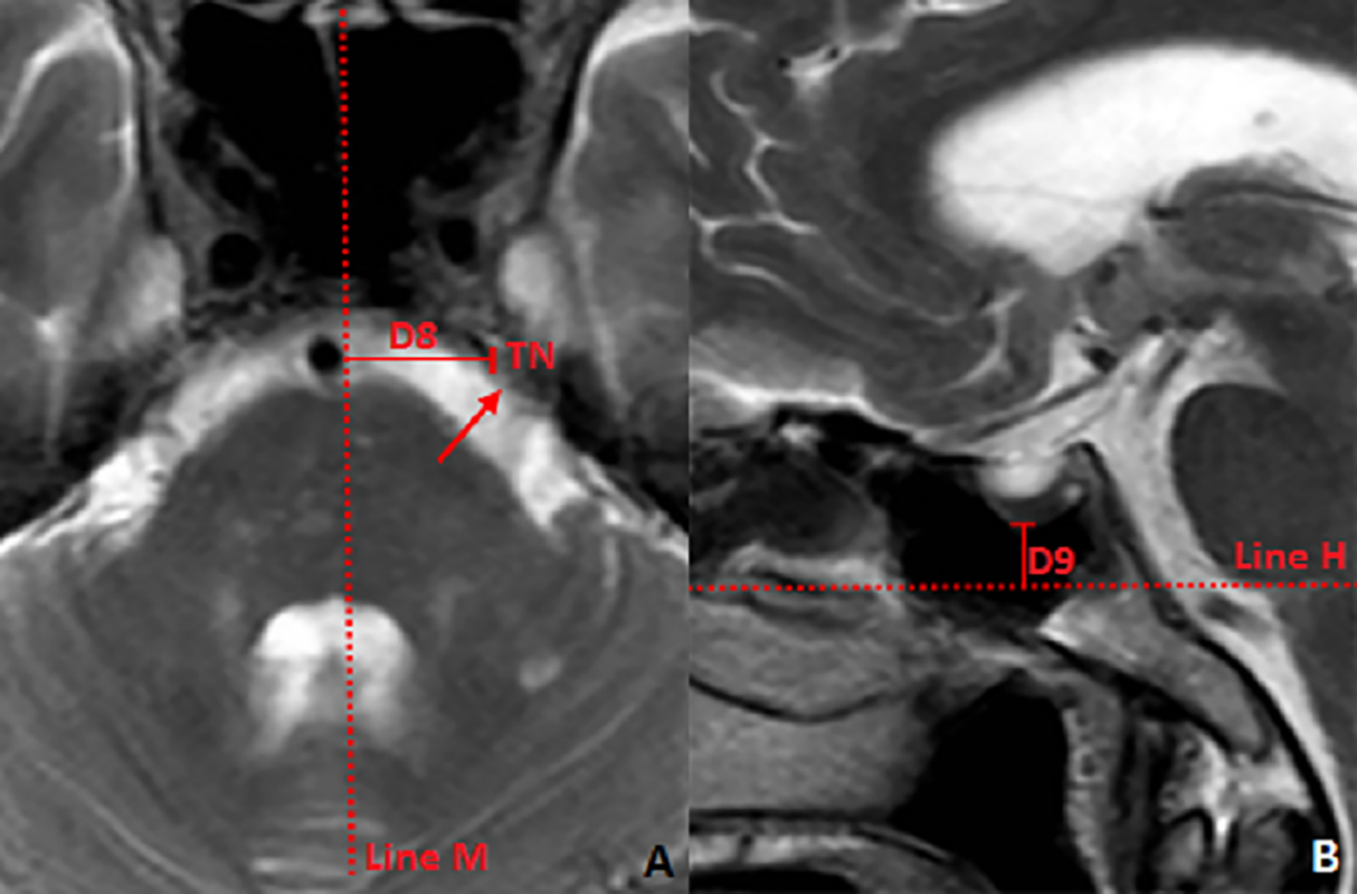
Location of trigeminal nerve in the MR image. **9A**: Distance from the initial part of the trigeminal nerve to the mid-sagittal line; **Line M:** mid-sagittal line; **TN**: trigeminal nerve; **D8:** distance between the root of the trigeminal nerve and the mid-sagittal line. **9B: Line S:** Position of the axial plane in plane 8A; **PG:** the pituitary gland; **D9:** the distance between the axial plane through the root of trigeminal nerve and lowest, central point of the SF.

**Fig 10:**
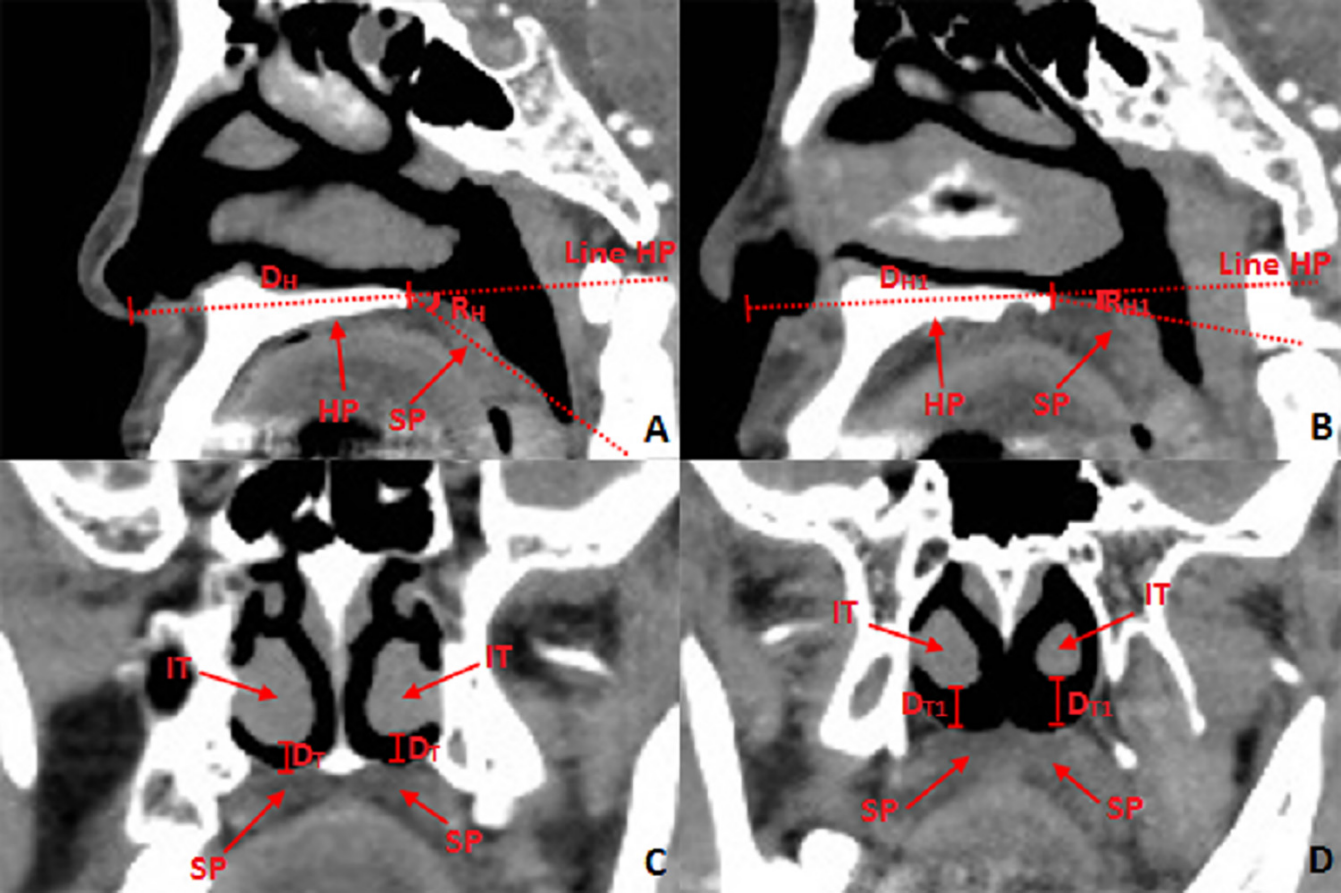
Location and measurement of soft palate in the view of endoscopic trans-nasal surgery. **10A:** Sagittal middle plane: **D**_**H**_: Distance between the nostril and the anterior part of the soft palate. **R**_**H**_: The angle between horizontal direction of soft palate and the direction of the soft palate. **Line HP:** The line in accordance with the horizontal direction of the hard palate. **10B:** Sagittal plane 10 mm right of mid-sagittal plane. **D**_**H1**_: Distance between the nostril and the anterior part of the soft palate. **R**_**H1**_: The angle between horizontal direction of soft palate and the direction of the soft palate. Line HP: **10C:** Coronal plane through the anterior point of soft palate. **D**_**T**_: Vertical distance between the inferior turbinate and soft palate in the coronal plane through the anterior part of the soft palate; **D**_**T1**_: Vertical distance between the inferior turbinate and soft palate; **IT:** inferior turbinate; **SP:** soft palate. **10D:** Coronal plane through the posterior point of inferior turbinate. **D**_**T1**_: Vertical distance between the inferior turbinate and soft palate.

The following axial planes crossing the surgical landmarks relevant to the trans-nasal trans-sphenoid approach were chosen: the central lowest point of the SF, the apertura sinus sphenoidalis, upper aspect of the foramen lacerum, and the superior margin of the nostril. These landmarks were used to determine the positions of the important neurovascular structures and thereby define the safe corridor for the penetration of the clivus (Fig 7A). Further, we also made the measurements of the distance between the following points: between the ICA and the mid-sagittal line and between the sulcus of the inferior petrosal sinus and the mid-sagittal line in these planes (Fig 7B–7E); between the foramen jugulare and the mid-sagittal line in the coronal plane through the foramen jugulare; and between the foramen jugulare to the mid-sagittal line of the SF (Fig 7F). The dry skull specimens were split along the coronal plane passing through the arcus superciliaris and internal occipital protuberance to reveal the inner surface of the basis cranii and determine the position of the sulcus for inferior petrosal sinus (Fig 8). In addition, the distance from the origin of the trigeminal nerve to the mid-sagittal line was retrieved from the coronal MR images (Fig 9). Furthermore, we identified the location of the soft palate in the view of the endoscopic trans-nasal trans-sphenoid surgery (Fig 10).

### Statistical analysis

Student *t* test was used to determine the significance of the difference between the various parameters. The statistical analysis was performed using the SPSS Inc. Released 2007. SPSS for Windows, Version 16.0 (SPSS Inc., Chicago, IL).

## Results

MPR of the CTA images enabled the identification and visualization of the entire clivus in the bone window. The clivus cave was identified in the CT images of all the specimens with sphenoid sinus of types IVa and IVb, defined according to the classification system proposed by Güldner et al [5]; these specimens comprised 70.9% (156 of 220 individuals) of those examined in this study. On compilation, the imaging data revealed that the length of the clivus (Lc) was 43.45±2.43 mm. The angle of the clivus (Rc) measured from the CT images was 67.41 ±1.98, which did not differ significantly from that measured from the specimens (66.92 ±2.03). This indicated the reliability of the measurements obtained from the CT images. The thickness of the clivus at various points is listed in Table 1. The horizontal distance was 9.67±1.02 mm between the lowest, central point of the SF and the posterior boundary of the clivus (D_A_) The distance between the posterior part of the middle turbinate and the anterior margin of the foramen magnum (D_N_) was 47.16±2.14 mm and 46.56±2.23 mm, that between the foramen jugulare and the mid-sagittal line (D_F_) was 21.45±0.34 mm and 21.01±0.33 mm, and that between the root of trigeminal nerve and the mid-sagittal line (D8) was 14.98±1.12 mm and 15.02±1.09 mm on the right and left sides, respectively. The angle between the line from the posterior part of the MT to the anterior part of the foramen magnum and the horizontal plane (R_N_) was 18.12 ±1.51. The distance between the coronal plane through the upper edge of foramen lacerum and the lowest point of the SF was 10.83±0.82 mm, and the axial plane through the root of trigeminal nerve (D9) was 8.41±1.48 mm inferior to the lowest, central point of SF. The distance between the posterior part of the IT and PA (D_PA_) was 31.31±1.84 mm and 32.44±1.93 mm, and the angle between the line from the posterior part of the IT to the PA and the horizontal plane (R_A_) was 31.25 ±2.51. In the axial plane through the PA, the distance between ICA and the mid-sagittal line was 13.12±1.73 mm, and the distance was 9.94±1.43 mm in the axial plane 10 mm superior to the PA and 28.12±2.73 mm in the axial plane 10 mm inferior to the PA.

**Table 1:** Thickness of clivus (mm).

Parameter	Mean	SD	Range
**T1**_**M**_	11.34	0.91	8.47~12.92
**T1**_**R**_	11.85	1.74	7.92~13.72
**T1**_**L**_ *	11.86	1.67	8.01~13.42
**T2**_**M**_	5.34	0.45	3.98~6.62
**T2**_**R**_	7.53	0.67	5.09~8.63
**T2**_**L**_ **†**	7.61	0.69	5.35~9.01

**T1**_**M**_ **, T1**_**R**_ **, T1**_**L**_: The thickness of the middle portion of the clivus in the middle, right, and left.

**T2**_**M**_ **T2**_**R**_ **T2**_**L**_: The thickness of inferior portion of clivus in the middle, right, and left.

* No significant difference was noted between the right (left) side of clivus and the middle part of clivus.

† significant difference was noted between the right (left) side of clivus and the lower part of clivus.

No significant difference was found in these parameters between the male and female subjects.

The distance between the ICA and the mid-sagittal line in different planes and that between the medial margin of sulcus for inferior petrosal sinus and mid-sagittal line in different planes are provided in Tables 2 and 3, respectively. The distance between the medial margin of sulcus for inferior petrosal sinus and mid-sagittal measured in specimen are provided in Table 4. The thickness of the soft palate was 0.97±0.21 mm at the middle and 1.34±0.27 mm in the right (left) part at the position of the anterior part. The locations of the soft palate in the procedure of endoscopic trans-nasal trans-sphenoid procedure are provided in Table 5.

**Table 2:** Distance between the medial wall of the ICA and mid-sagittal line (mm).

parameter		mean	SD	Range
**D1**	Right	10.36	0.45	9.85~11.72
	Left	10.57	0.51	9.12~11.98
**D2**	Right	10.76	0.43	9.46~12.12
	Left	10.19	0.34	8.95~11.87
**D3**	Right	10.76	0.44	9.84~12.53
	Left	10.44	0.41	9.57~11.23
**D4**	Right	15.01	0.43	13.65~17.12
	Left	14.89	0.48	13.01~16.82

**D1**: Distance between the medial wall of the ICA and mid-sagittal line in plane B

**D2**: Distance between the medial wall of the ICA and mid-sagittal line in plane C

**D3**: Distance between the medial wall of the foramen lacerum and mid-sagittal line in plane D

**D4**: Distance between the medial wall of ICA and mid-sagittal line in plane E

**Table 3:** Distance between sulcus of the inferior petrosal sinus and the mid-sagittal line (mm).

Parameter		Mean	SD	Range
**D5**	Right	10.55	0.45	8.98~12.39
	Left	10.49	0.39	9.01~12.32
**D6**	Right	11.89	0.45	9.85~14.21
	Left	11.83	0.38	10.31~13.24
**D7**	Right	14.16	0.49	12.45~16.81
	Left	14.08	0.48	12.21~17.04

**D5:** Distance between the sulcus of the inferior petrosal sinus and the mid-sagittal line in plane C

**D6:** Distance between the sulcus of the inferior petrosal sinus and the mid-sagittal line in plane D

**D7:** Distance between the sulcus of the inferior petrosal sinus and the mid-sagittal line in plane E

**Table 4:** Distance between sulcus for inferior petrosal sinus and the sagittal midline in specimen (mm).

parameter		Mean	SD	Range
**D5′**	Right	10.67	0.55	9.01~12.39
**D6′**	Right	11.99	0.53	9.77~13.91
**D7’**	Right	14.33	0.59	12.15~16.51

*No significant difference was noted between L5 and L5′, L6 and L6′, and L7 and L7′, as shown by p < 0.05 in Student’s *t*-test.

**Table 5:** The location of the soft palate in the view of endoscopic trans-nasal surgery to clivus region.

parameter	Mean	SD	Range
**D**_**H**_ **(right, mm)**	**58.31**	**1.13**	**54.12~61.88**
**R**_**H**_ **(right, degree)**	**47.87**	**2.09**	**40.54~54.32**
**D**_**H1**_ **(right, mm)**	**50.18**	**1.28**	**45.67~55.31**
**R**_**H1**_ **(right, degree)**	**18.81**	**1.65**	**12.45~23.99**
**D**_**T**_ **(right, mm)**	**5.23**	**0.67**	**3.91~7.31**
**D**_**T1**_ **(right, degree)**	**9.65**	**0.99**	**6.75~12.54**

**D**_**H**_: Distance between the nostril and the anterior part of the soft palate in sagittal middle plane.

**R**_**H**_: The angle between horizontal direction of soft palate and the direction of the soft palate in mid-sagittal line.

**D**_**H1**_: Distance between the nostril and the initial point of the soft palate in the position 10 mm right to the mid-sagittal plane.

**R**_**H1**_: The angle between horizontal direction of soft palate and the direction of the soft palate in the position 10 mm right to the mid-sagittal plane.

**D**_**T**_: Vertical distance between the inferior turbinate and soft palate in the coronal plane through the anterior part of the soft palate.

**D**_**T1:**_ Vertical distance between the inferior turbinate and soft palate in the coronal plane through the posterior part of the inferior turbinate.

## Discussion

Several operative procedures can be used to approach the clivus region, including the subtemporal, suboccipital, extrapolar, orophargngeal, and transnasal–sphenoidal approaches. None of these approaches can be considered as universally applicable since there is considerable variation among individuals and clinical conditions [6–9]. However, the rapid advances in the endoscopic technology have led to the increased use of the nasal trans-sphenoidal approach for the resection of sellar tumors invading the walls of the sella turcica [1, 4]. Endoscopy offers the advantages of visualization of deeper structures under illumination, a clear surgical field, maneuverability of the instruments, and ability for multi-perspective observation; these advantages overcome the drawbacks of poor visualization and the technical difficulty associated with the use of the traditional operating microscope, making it possible to approach the clivus [10]. However, trans-nasal trans-sphenoid tranclivus surgery remains challenging because of the deep position of the clivus itself, the limited operational space, the lack of three-dimension visualization, the distortion of the two-dimensional images, and the high risk of injury to important neurovascular structures such as the ICA and sulcus of the inferior petrosal sinus [11]. This highlights the need for a comprehensive anatomical understanding for the identification of consistent, stationary landmarks that will help define a safe and accurate operative region. Accordingly, this study was undertaken with the view to measure the dimensions of the clivus from various stationary anatomical landmarks and its relation to important neurovascular structures in order to determine a safe corridor for approaching the clivus.

### The anatomical measurement of clivus

The clivus is located posterior to the sphenoid cavity, which can alter in shape depending on the degree of pneumatization of the sphenoid sinus. An important landmark relevant to surgeries involving the clivus is the mid-sagittal line, which can be identified by the central, lowest point of the SF and the midpoint of the optic recess [12]. The sphenoid sinus was classified into 4 types as per the criteria put forth by Güldner: type I, in which the sphenoid sinus was completely missing or minimal and types II, III, and IV, in which the posterior wall of the sphenoid sinus was positioned in front of the anterior wall of the sella, between the anterior and posterior walls of the sella, and behind the posterior wall of the sella, respectively [5]. Our findings indicated that the occurrence of the clivus recess is closely related to the degree of the pneumatization of the sphenoid sinus. Accordingly, the gasification of the clivus depended on both sides of the sphenoid sinus rather than the mid-sagittal plane, in accordance with the latest classification of pneumatization.

With regard to the long axis of the clivus, the length and inclination angle did not show any significant difference with age and sex. Since the plotting of the horizontal lines in the CT images may be influenced by the position of the subject’s body in the CT scanner, the obtained results were compared with the measurements made in skull specimens serving as the controls. The results of the comparison showed no significant difference between the measurements obtained in both analyses. The thickness of the clivus in the superior portion varied with the degree of pneumatization of the sphenoid sinus, while that in the middle and inferior portions remained relatively consistent. Our findings showed that during surgeries involving the brain stem, the depth of penetration of the middle portion of the clivus is about 9.67±1.02 mm, and that proper equipment should be selected for this procedure. In the inferior portion of the clivus, the thickness varied significantly between the sides and middle portions. This may be attributed to the shape of the clivus, which in accordance with the shape of the brain stem, is curved. Therefore, the depth of penetration of the inferior portion of the clivus are about 5.34±0.45 mm and 7.53±0.67 mm in the middle and the side portions of the clivus, respectively.

### Safe corridor for surgical access to the clivus

Bony landmarks are critical to endoscopic trans-nasal trans-sphenoidal surgery. Some of these landmarks are the lowest, central point of the SF, which is a stable, visible point from the sphenoid cavity identifiable during the surgery [13–14], as well as the inferior turbinate, inferior turbinate, and posterior nares [15]. Thus, the horizontal distance between the lowest, central point of the SF and the posterior edge of clivus was 9.67±1.02 mm. This implied that although the thickness of the clivus varies at different portions, the surgeon can fully remove the clivus bone at a distance of 9.67±1.02 mm from the lowest central point of SF in the posterior direction. Further, the anterior margin of the foramen magnum would be 41.38±2.54 mm from the posterior nares, at an angle of 17.85 ±2.35 from the horizontal plane.

During transclivus surgery, it is important to avoid injury to important adjacent structures such as the optic nerve, subpetrosal sinuses, and ICA. The ICA is closely related to the transclivus surgery. Bouthillier et al [16] divided the ICA it into 7 segments, namely, the cervical, petrous, lacerum, cavernous, clinoid, ophthalmic, and communicating segments. During the transclivus surgery, care should be taken to avoid injury to the lacerum and cavernous segments of the clivus. Further, in the plane through the lowest, central point of the SF, the width of the penetration in the posterior wall of the sphenoid sinus should be limited to within 10.36±0.45 mm from the mid-sagittal line. On classifying the cavernous segment of the ICA into types Z, S, and R, as per the criteria defined by Wang [17], the straight posterior portion located on the posterior lateral wall of the sphenoid sinus in type Z was found in the coronal plane passing through the lowest, central point of the SF. We noted that in 67.6% of the subjects, the cavernous segment of the ICA was of the Z type, which renders our findings particularly useful because they suggest that the position of the ICA should be carefully accounted for during penetration in the superior position. In cases where the cavernous segment of the ICA was of types R or S, the position of the segment was horizontal and the posterior straight segment of ICA was placed much lower, thereby reducing the risk of injury to the ICA during the penetration of the clivus.

Petrous apex is a key anatomic structure involved in trans-nasal surgery; Jamie et al have divided the PA into superior and inferior portions, making the case that inferior PA lesions were best treated endoscopically, while superior lesions are best treated by an open approach [18]. Our measurements related to the PA and the paraclival carotid are complementary to this notion. In our study, we found that at the position 10 mm superior to PA, the distance between the ICA and the mid-sagittal line was 9.94±1.43 mm while in the position 10 mm inferior to PA, the distance between ICA and the mid-sagittal line was 28.12±2.73 mm, implying that the operative width on the clivus was restricted in the position superior to the PA due to the close distance between the bilateral side of the ICA. However, no restriction in the position inferior to the PA which is suitable to the endoscopic surgery. Moreover, we first located the PA by the bony landmarks-inferior turbinate; our study indicated the PA is 31.31±1.84 mm from the posterior point of the IT, and the direction is 31.25 ±2.51 from the horizontal line.

For the endoscopic treatment of lesions located around the mid-sagittal line superior and inferior of the PA, the penetration width must be restricted according to the following observations regarding the safe corridor of clivus penetration: The length of the sulcus of the inferior petrosal sinus, first measured from the CT image, was verified using the measurements made from the skull specimens and found to show no significant difference between the two sets of measurements. To avoid injury to the subpetrosal sinuses, the width of the penetration of the clivus in the posterior wall of the sphenoid sinus should be limited within 10.36±0.45 mm from the mid-sagittal line in the plane passing through the lowest, central point of the SF; less than 10.76±0.43mm from the mid-sagittal line, in the plane passing through the aperture of the sphenoid sinus; and 10.55±0.45 mm from the mid-sagittal line. Further, the width of the penetration in the posterior wall of the sphenoid sinus should be limited to less than 10.76±0.44 mm from the midline and within 11.89±0.45 mm during penetration of the posterior aspect of the clivus in the plane passing through the foramen lacerum, which is 10.83±0.82 mm below the lowest, central point of the SF measured in this study. In the plane passing through the upper margin of the posterior nares, the width of penetration in the posterior wall of the sphenoid sinus should be limited to 15.01±0.43 mm and 14.16±0.49 mm from the mid-sagittal line and the posterior wall of the clivus, respectively. In the plane of the foramen magnum, the width of the penetration should be within 21.45±0.34 mm and 21.01±0.33 mm on the right and left sides, respectively, to avoid injury to the internal jugular vein. Further, it is necessary to consider the position of the origin of the trigeminal nerve during extended trans-sphenoid surgery; the origin of trigeminal nerve from the brain stem was 14.98±1.12 mm on the right side and 15.02±1.09 mm from the mid-sagittal line, and the axial through it passed about 3 mm above the lowest, central point of the SF.

### Soft palate in endoscopic trans-nasal surgery to clivus region

During the endoscopic trans-nasal trans-sphenoid surgery with inferiorly pointed lesions and lesions with a steep clival angle, the protection of soft palate is an important concern, since injury of the soft palate can result in dysphonia as well as infection of the oral cavity [19]. The connection point between the soft palate and hard palate is not easily detected since both of them are covered with the mucous membrane; therefore, we measured the distance between the nostril to the anterior point of the soft palate in the mid-sagittal plane and the plane at 10 mm to the right (left); these distances were 58.31±1.13 mm and 50.18±1.28 mm, respectively. The anterior point of soft palate was also located at 5.23±0.67 mm and 9.65±0.99 mm in the anterior part of soft palate and posterior part of inferior turbinate The thickness of the soft palate were 0.97±0.21 mm at the middle and 1.34±0.27 mm at the right and left sides. From the anterior point of the soft palate, the angle to the downward should be restricted between 47.87±2.09° and 18.81±1.65°.

Conclusively, our findings are expected to change the practice of the endoscopic clivus surgery in the following aspects. Firstly, the safe corridor facilitates endoscopic penetration of the clivus into lesions around the mid-sagittal line even at the position superior to the PA. Additionally, the study results provide a novel method for the location of PA and approaching via the posterior part of MT instead of totally removing the MT first. Furthermore, the findings also allow for the identification and location of the soft palate by surgical landmarks in order to avoid injuring it.

## Conclusions

Thus, our findings provide guidelines that define a safe corridor to approach the clivus via the endoscopic transclivus approach, on the basis of some fixed anatomical landmarks. The procedure is otherwise technically challenging because of the high risk of injury to important neurovascular structures. We believe that the results obtained in this study will enhance the surgical safety of this procedure and aid in the choice of the appropriate endoscopic equipment for the procedure.

## Supporting Information

S1 Table Data of length of the clivus (Lc) (measured by CT). (DOCX)

S2 Table Data of angle of the clivus (Rc) measured in specimen. (DOCX)

S3 Table Data of angle of the clivus (Rc) measured by CT. (DOCX)

S4 Table Data of distance between the root of trigeminal nerve and the mid-sagittal line (D8) (measured by MRI). (DOCX)
