# Supplementary material for: Safe Corridor to Access Clivus for Endoscopic Trans-Sphenoidal Surgery: A Radiological and Anatomical Study
Source: PLoS One. 2015 Sep 14;10(9):e0137962. doi: 10.1371/journal.pone.0137962 (PMC4569549; doi:10.1371/journal.pone.0137962)
Supplement: S1 Table — (DOCX) [file pone.0137962.s001.docx]

**S1 Table. Data of length of the clivus (Lc) (measured by CT)**

| Mean (mm) | | 43.4476 | |  |  |  |  |
| --- | --- | --- | --- | --- | --- | --- | --- |
| SD (mm) | | 2.4296 | |  |  |  |  |
| Minimum (mm) | | 36.86 | |  |  |  |  |
| Maximum (mm) | | 49.88 | |  |  |  |  |
| N | | 220 | |  |  |  |  |
| Data (mm) |  | |  | |  |  |  |
| \| 39.72 \| 47.61 \| 42.87 \| 43.15 \| 44.83 \| 43.47 \| 41.92 \| \| --- \| --- \| --- \| --- \| --- \| --- \| --- \| \| 44.90 \| 42.15 \| 42.23 \| 41.76 \| 44.76 \| 42.41 \| 45.39 \| \| 44.19 \| 43.79 \| 44.95 \| 42.80 \| 40.51 \| 36.98 \| 39.54 \| \| 49.01 \| 43.76 \| 41.51 \| 44.97 \| 49.88 \| 43.62 \| 42.78 \| \| 40.66 \| 42.62 \| 42.73 \| 44.07 \| 41.32 \| 42.10 \| 42.84 \| \| 45.13 \| 39.01 \| 44.07 \| 43.33 \| 41.38 \| 43.51 \| 42.70 \| \| 41.98 \| 42.45 \| 46.31 \| 42.06 \| 41.64 \| 44.60 \| 44.04 \| \| 41.98 \| 43.44 \| 43.42 \| 43.95 \| 44.85 \| 42.79 \| 48.06 \| \| 43.04 \| 44.01 \| 43.17 \| 45.59 \| 41.95 \| 47.12 \| 45.10 \| \| 44.72 \| 40.82 \| 42.69 \| 44.11 \| 43.09 \| 41.90 \| 47.11 \| \| 44.42 \| 40.41 \| 43.36 \| 40.12 \| 44.38 \| 41.89 \| 43.92 \| \| 46.54 \| 45.78 \| 43.49 \| 42.24 \| 39.32 \| 43.71 \| 43.88 \| \| 45.26 \| 38.43 \| 43.42 \| 44.81 \| 46.90 \| 39.83 \| 49.78 \| \| 44.21 \| 43.48 \| 43.67 \| 43.19 \| 43.57 \| 43.25 \| 42.26 \| \| 41.56 \| 43.24 \| 43.96 \| 40.23 \| 40.00 \| 42.63 \| 42.90 \| \| 46.54 \| 42.19 \| 41.73 \| 47.81 \| 46.99 \| 48.56 \| 42.06 \| \| 48.76 \| 43.13 \| 45.99 \| 39.60 \| 43.75 \| 43.23 \| 45.56 \| \| 43.60 \| 46.01 \| 44.45 \| 41.41 \| 43.35 \| 42.26 \| 44.41 \| \| 43.64 \| 47.34 \| 39.74 \| 45.21 \| 40.21 \| 42.81 \| 40.12 \| \| 43.59 \| 39.22 \| 39.14 \| 44.09 \| 43.98 \| 47.99 \| 42.34 \| \| 41.76 \| 40.70 \| 42.51 \| 43.46 \| 45.31 \| 44.60 \| 42.36 \| \| 42.95 \| 42.88 \| 43.38 \| 42.83 \| 41.01 \| 46.71 \| 46.41 \| \| 48.45 \| 40.48 \| 37.45 \| 43.52 \| 41.23 \| 36.86 \| 40.31 \| \| 43.45 \| 44.31 \| 43.55 \| 43.60 \| 46.69 \| 44.16 \| 44.33 \| \| 41.00 \| 44.54 \| 41.32 \| 37.05 \| 43.90 \| 48.01 \| 47.50 \| \| 43.98 \| 43.07 \| 41.31 \| 43.85 \| 39.92 \| 41.53 \| 45.62 \| \| 41.46 \| 45.42 \| 45.84 \| 45.15 \| 45.92 \| 43.41 \| 42.05 \| \| 43.21 \| 45.65 \| 44.14 \| 44.56 \| 43.81 \| 42.72 \| 48.21 \| \| 43.01 \| 41.54 \| 46.21 \| 41.39 \| 43.55 \| 42.54 \| 48.63 \| \| 43.70 \| 47.21 \| 38.01 \| 44.79 \| 46.05 \| 44.87 \| 47.54 \| \| 43.16 \| 45.74 \| 40.61 \| 38.66 \| 45.80 \| 45.23 \| 42.39 \| \| 44.68 \| 42.15 \| 43.83 \|  \|  \|  \|  \| | | | | | | | |
