# Supplementary material for: Safe Corridor to Access Clivus for Endoscopic Trans-Sphenoidal Surgery: A Radiological and Anatomical Study
Source: PLoS One. 2015 Sep 14;10(9):e0137962. doi: 10.1371/journal.pone.0137962 (PMC4569549; doi:10.1371/journal.pone.0137962)
Supplement: S2 Table — (DOCX) [file pone.0137962.s002.docx]

**S2 Table. Data of the angle of the clivus (Rc) measured in specimen**

| Mean (mm) | 66.9200 |  |  |  |
| --- | --- | --- | --- | --- |
| SD (mm) | 2.0313 |  |  |  |
| Minimum (mm) | 63.15 |  |  |  |
| Maximum (mm) | 73.00 |  |  |  |
| N | 10 |  |  |  |
| Data |  |  |  |  |
| \| 65.21 \| 68.09 \| 64.75 \| 63.15 \| 68.67 \| 70.1 \| 66.98 \| \| --- \| --- \| --- \| --- \| --- \| --- \| --- \| \| 67.38 \| 67.45 \| 67.42 \|  \|  \|  \|  \| | | | | |
