# Supplementary material for: Safe Corridor to Access Clivus for Endoscopic Trans-Sphenoidal Surgery: A Radiological and Anatomical Study
Source: PLoS One. 2015 Sep 14;10(9):e0137962. doi: 10.1371/journal.pone.0137962 (PMC4569549; doi:10.1371/journal.pone.0137962)
Supplement: S3 Table — (DOCX) [file pone.0137962.s003.docx]

**S3 Table. Data of the angle of the clivus (Rc) measured by CT**

| Mean (mm) | 67.4149 |  |  |  |
| --- | --- | --- | --- | --- |
| SD (mm) | 1.9834 |  |  |  |
| Minimum (mm) | 61.99 |  |  |  |
| Maximum (mm) | 72.29 |  |  |  |
| N | 220 |  |  |  |
| Data |  |  |  |  |
| \| 69.12 \| 65.35 \| 71.45 \| 65.99 \| 68.21 \| 67.34 \| 67.41 \| \| --- \| --- \| --- \| --- \| --- \| --- \| --- \| \| 69.56 \| 66.45 \| 69.45 \| 71.94 \| 69.21 \| 64.21 \| 67.1 \| \| 67.52 \| 65.23 \| 66.78 \| 66.31 \| 69.09 \| 67.97 \| 67.78 \| \| 70.39 \| 65.89 \| 66.01 \| 68.5 \| 69.42 \| 65.41 \| 67.14 \| \| 67.21 \| 67.42 \| 67.42 \| 66.88 \| 67.75 \| 65.49 \| 68.35 \| \| 68.05 \| 69.34 \| 67.95 \| 65.51 \| 68.8 \| 68.7 \| 68.41 \| \| 68.58 \| 69.63 \| 67.46 \| 67.45 \| 69.68 \| 69.05 \| 65.31 \| \| 68.67 \| 64.45 \| 66.83 \| 65.28 \| 67.49 \| 69.32 \| 67.31 \| \| 68.94 \| 65.14 \| 66.91 \| 66.74 \| 67.42 \| 67.91 \| 64.75 \| \| 67.03 \| 63.34 \| 69.54 \| 67.93 \| 65.39 \| 68.32 \| 65.81 \| \| 67.4 \| 67.97 \| 65.1 \| 65.94 \| 64.54 \| 68.12 \| 66.28 \| \| 69.78 \| 72.29 \| 72.03 \| 65.29 \| 67.34 \| 66.97 \| 64.7 \| \| 67.44 \| 68.44 \| 67.1 \| 68.1 \| 65.44 \| 67.84 \| 67.79 \| \| 67.29 \| 67.36 \| 66.21 \| 67.53 \| 67.16 \| 63.78 \| 70.04 \| \| 71.56 \| 66.01 \| 65.78 \| 67.12 \| 62.59 \| 64.61 \| 65.54 \| \| 67.1 \| 68.99 \| 67.22 \| 63.01 \| 69.61 \| 68.06 \| 66.94 \| \| 70.87 \| 69.89 \| 64.81 \| 70.01 \| 65.71 \| 65.19 \| 70.54 \| \| 66.52 \| 67.39 \| 64.39 \| 67.48 \| 70.59 \| 67.4 \| 67.26 \| \| 65.63 \| 64.57 \| 67.23 \| 64.3 \| 70.88 \| 68.61 \| 62.55 \| \| 68.01 \| 70.31 \| 68.68 \| 66.61 \| 65.36 \| 66.22 \| 66.55 \| \| 68.03 \| 64.99 \| 64.19 \| 70.24 \| 65.42 \| 68.82 \| 67.01 \| \| 68.72 \| 64.06 \| 66.39 \| 69.71 \| 68.75 \| 67.97 \| 69.99 \| \| 66.04 \| 67.33 \| 67.25 \| 65.81 \| 67.61 \| 66.04 \| 67.65 \| \| 67.91 \| 61.99 \| 64.11 \| 68.58 \| 70.81 \| 68.78 \| 66.36 \| \| 69.47 \| 67.74 \| 69.15 \| 68.52 \| 68.78 \| 67.6 \| 67.34 \| \| 66.42 \| 66.06 \| 66.72 \| 71.21 \| 67.19 \| 70.7 \| 67.23 \| \| 68.55 \| 66.81 \| 70.7 \| 67.9 \| 65.86 \| 70.51 \| 67.51 \| \| 67.35 \| 66.58 \| 66.33 \| 70.68 \| 67.81 \| 66.04 \| 64.24 \| \| 67.71 \| 64.3 \| 65.01 \| 69.99 \| 68.97 \| 67.41 \| 66.99 \| \| 67.63 \| 70.11 \| 67.16 \| 65.12 \| 66.66 \| 67.4 \| 67.68 \| \| 68.18 \| 69.91 \| 63.01 \| 66.71 \| 70.15 \| 63.67 \| 67.39 \| \| 70.02 \| 68.7 \| 71.67 \|  \|  \|  \|  \| | | | | |
