# Supplementary material for: Safe Corridor to Access Clivus for Endoscopic Trans-Sphenoidal Surgery: A Radiological and Anatomical Study
Source: PLoS One. 2015 Sep 14;10(9):e0137962. doi: 10.1371/journal.pone.0137962 (PMC4569549; doi:10.1371/journal.pone.0137962)
Supplement: S4 Table — (DOCX) [file pone.0137962.s004.docx]

**S4 Table. Data of distance between the root of trigeminal nerve and the mid-sagittal line (D8) (measured by MRI)**

| Mean (mm) | 14.9804 |  |  |  |  |
| --- | --- | --- | --- | --- | --- |
| SD (mm) | 1.1201 |  |  |  |  |
| Minimum (mm) | 12.23 |  |  |  |  |
| Maximum (mm) | 18.06 |  |  |  |  |
| N | 50 |  |  |  |  |
| \| 14.82 \| 15.21 \| 14.9 \| 14.31 \| 14.97 \| 14.46 \| 14.87 \| \| --- \| --- \| --- \| --- \| --- \| --- \| --- \| \| 14.79 \| 14.51 \| 12.23 \| 14.19 \| 15.88 \| 16.99 \| 15.14 \| \| 15.55 \| 15.43 \| 14.99 \| 12.56 \| 14.9 \| 13.93 \| 14.99 \| \| 14.33 \| 16.65 \| 14.08 \| 15.43 \| 16.48 \| 13.2 \| 16.59 \| \| 17.31 \| 14.65 \| 14.73 \| 15.29 \| 14.56 \| 15.38 \| 14.01 \| \| 13.55 \| 15.03 \| 18.06 \| 15.65 \| 15.22 \| 13.72 \| 16.13 \| \| 14.26 \| 15.01 \| 13.44 \| 16.04 \| 15.92 \| 15.61 \| 14.55 \| \| 14.52 \|  \|  \|  \|  \|  \|  \| | | | | | |
